# Supplementary material for: Comparative Transcriptomic Analysis Uncovers Genes Responsible for the DHA Enhancement in the Mutant Aurantiochytrium sp
Source: Microorganisms. 2020 Apr 7;8(4):529. doi: 10.3390/microorganisms8040529 (PMC7232246; doi:10.3390/microorganisms8040529)
Supplement: Supplementary file 1 [file microorganisms-08-00529-s001.pdf]

Table 1S. List of mRNA primers

mRNA primer	Sequence
Enoyl-(Acyl carrier protein) reductase gene-F	5'-CTCGCGTTGTGATTGCTGAT-3'
Enoyl-(Acyl carrier protein) reductase gene-R	5'-CTTGCTGAGGGTCCTAATGG-3'
CoA-transferase family III gene-F	5'-TTTTACGGCCTTCTGGTGTC-3'
CoA-transferase family III gene-R	5'-GCATGATGCTGGCTGATTTT-3'
Acyltransferase gene-F	5'-AGTTCTGCACTGGTCCTACA-3'
Acyltransferase gene-R	5'-ATTCTCGCAGTAAACCGTAG-3'
Methyltransferase gene-F	5'-CGTTATTACGCAGCCCAAGA-3'
Methyltransferase gene-R	5'-TCCAACAAGACCAGCATCAA-3'
Dehydratase family gene-F	5'-TTGTTGATGCTGGTCTTGTT-3'
Dehydratase family gene-R	5'-AATAGAGTCGGCGATGATGT-3'
